# Adult mental health provision in England: a national survey of acute day units

**DOI:** 10.1186/s12913-019-4687-8

**Published:** 2019-11-21

**Authors:** Danielle Lamb, Michael Davidson, Brynmor Lloyd-Evans, Sonia Johnson, Samira Heinkel, Thomas Steare, Vanessa Pinfold, Scott Weich, Nicola Morant, James Kirkbride, Louise Marston, Alastair Canaway, Jason Madan, David Osborn

**Affiliations:** 1 University College London, London, England; 2 McPin Foundation, London, England; 3 University of Sheffield, Sheffield, England; 4 University of Warwick, Coventry, England

**Keywords:** Mental health, Psychiatry, Acute day unit, Alternative to admission, Survey

## Abstract

**Background:**

Acute Day Units (ADUs) exist in some English NHS Trusts as an alternative to psychiatric inpatient admission. However, there is a lack of information about the number, configuration, and functioning of such units, and about the extent to which additional units might reduce admissions. This cross-sectional survey and cluster analysis of ADUs aimed to identify, categorise, and describe Acute Day Units (ADUs) in England.

**Methods:**

English NHS Mental Health Trusts with ADUs were identified in a mapping exercise, and a questionnaire was distributed to ADU managers. Cluster analysis was used to identify distinct models of service, and descriptive statistics are given to summarise the results of the survey questions.

**Results:**

Two types of service were identified by the cluster analysis: NHS (*n* = 27; and voluntary sector services (*n* = 18). Under a third of NHS Trusts have access to ADUs. NHS services typically have multi-disciplinary staff teams, operate during office hours, offer a range of interventions (medication, physical checks, psychological interventions, group sessions, peer support), and had a median treatment period of 30 days. Voluntary sector services had mostly non-clinically qualified staff, and typically offered supportive listening on a one-off, drop-in basis. Nearly all services aim to prevent or reduce inpatient admissions. Voluntary sector services had more involvement by service users and carers in management and running of the service than NHS services.

**Conclusions:**

The majority of NHS Trusts do not provide ADUs, despite their potential to reduce inpatient admissions. Further research of ADUs is required to establish their effectiveness and acceptability to service users, carers, and staff.

## Background

The global socioeconomic burden of mental ill-health is estimated to be as high as that of cardiovascular diseases [1]. There are well-established challenges facing acute mental health care for people experiencing crises. These include: poor experience of services, lack of provision of recommended interventions, delays in accessing care, poor continuity of care, over-reliance on restriction orders, use of police for conveyance, overcrowding in Emergency Departments, and continuing issues with reduced bed capacity [2, 3]. This is a world-wide problem, and a range of reports have highlighted the need for better crisis care in the UK, including the recent Care Quality Commission report about mental health services [4], the Chief Medical Officer’s report in 2013 [5], the Crisis Care Concordat [6], and the final report by the Commission on Acute Adult Psychiatric Care [7].

Acute Day Units (ADUs) have the potential to address these challenges. These units offer intensive, short-term community responses to mental health crises, and aim to reduce costly and unpopular admissions, either avoiding them or facilitating early discharge. Existing crisis care provided by inpatient wards and Crisis Resolution Teams (CRTs) can be augmented by ADUs, which may be particularly helpful for people who are socially isolated or have poor social support, lack activities, or who would benefit from peer support or group interventions. Previous research reported that around 1 in 5 NHS mental health CRTs in England had access to ADUs within their catchment areas [8].

Non-residential day services have been a component of adult mental health services for decades, particularly across Europe [9]. Previously known as ‘day hospitals’, the intervention they offered have been varied, but typically involved longer periods of care than more recent incarnations of these units offer. The model for ADUs in the NHS has moved towards providing a shorter intervention, avoiding or shortening inpatient admission by supporting people in the acute phase of illness. In addition to NHS services, there are now many non-residential crisis services provided by voluntary sector organisations, which typically offer social interventions and support rather than medical or psychological treatment, for example, drop-in ‘crisis cafes’, though research of such services is lacking [10].

Cochrane systematic reviews have compared acute day hospitals to both outpatient and inpatient psychiatric care [11, 12]. The limited available evidence is heterogeneous in terms of study participants, design, and outcomes, making conclusions difficult. The most recent meta-analysis [12] involved ten randomised controlled trials conducted in the USA and Europe. It concluded that mental health day units were as effective as inpatient care in terms of readmission rates after discharge, employment, quality of life, and treatment satisfaction, but that more research was needed to establish the cost-effectiveness of such units.

The most recent British randomised controlled trial (RCT), involving one London ADU and three inpatient wards, is also promising, reporting that symptom improvement and satisfaction were greater at discharge in the ADU group [13]. This trial found that costs for ADU patients overall were greater than for inpatients, but this was largely due to mean ADU admissions being nearly twice as long as inpatient admission (55.7 ADU days vs. 30.5 inpatient days), with the cost per day of ADU treatment being only 70% that of inpatient care.

There is lack of more recent research about ADUs [12]. In the UK, this is likely to be due to the fact that while CRTs became mandatory with the 2000 NHS Plan [14], other acute community services such as crisis houses and ADUs were not established nationwide. A recent survey of CRTs found that just 22% (40/185) had access to an ADU, and we know from this research that implementation of acute services in practice is often highly variable and sub-optimal [8].

The Crisis Care Concordat [6] includes crisis care and acute day care within its domains, and ADUs address many of the ambitions of the NHS Five Year Forward View [14], including improvements in acute care, personalised care, empowerment and efficiency. ADUs have potential to be an important part of a well-developed crisis care system, offering user choice and greater possibilities for tailoring response to needs, but we currently lack clear evidence about how best to integrate them into contemporary systems.

We therefore aimed to identify and survey all ADUs in England in the NHS and voluntary sector, in order to: i) distinguish whether there are different service models; and ii) describe service delivery and organisation in ADUs nationally (applying any typology developed by aim i).

## Methods

### Design

An expert working group from the study team (comprising people with lived experience of using acute mental health services, clinicians, and researchers) constructed the 67-question survey, which covered the following areas (the full survey is available as a Additional file 2):

- Location and contact details
- Type of service (public sector, voluntary sector etc.)
- Funding
- Purpose of the service
- Joint working with other services
- Interventions provided
- Referral and discharge details
- Client group served
- Duration of care
- Service capacity and usage
- Staffing
- Service user involvement
- Service development

### Participants

ADUs were defined as non-residential services offering intensive treatment and care at a service site (i.e. not in people’s homes) to adults experiencing a mental health crisis. That is, people who would be considered for an acute psychiatric inpatient ward, or other alternatives to admission (including CRTs). Services were excluded which:

- Provide rehabilitation, rather than acute care;
- Work only with groups of service users who would not be considered for acute psychiatric hospital admission;
- Work primarily with populations other than people with mental illness (such as people with dementia, learning difficulties or primary drug or alcohol dependence disorders);
- Routinely work with service users for longer than three months;
- Do not accept referrals from the local CRTs.

To gain a comprehensive picture of services available, voluntary sector services meeting the criteria were included. Independent providers were not sought, because they are not available through NHS funding.

### Procedure

All NHS mental health trusts in England (*n* = 58) were contacted in August 2016 in the following ways: all England NHS Mental Health Trust websites were screened; local communications teams, Patient Advice and Liaison Service teams, Research and Development teams, Trust headquarters, local Acute Care Leads or other appropriate clinical staff were contacted by telephone and email; relevant professional organisations and networks (such as the Royal College of Psychiatrists Acute Care Network, and the MIND Acute Care Campaign) were contacted using Twitter, email and phone. Additionally, the CRT managers of all teams identified as having an ADU in the 2012 CRT Optimisation and Relapse Prevention (CORE) study survey [8] were contacted. Online searches were conducted for any voluntary sector services that met the inclusion criteria.

After screening and exclusion according to the criteria above, study researchers contacted the managers of the ADUs identified. Contact was made by telephone to explain the survey, answer any questions, and obtain email/postal addresses to send out information sheets.

Managers were able to nominate an alternative clinician (e.g. deputy manager, clinical lead), with appropriate knowledge of the ADU organisation and service delivery, to respond to the survey. Respondents were able to choose whether to complete the survey as a phone interview with a researcher or online using the secure UCL Opinio survey website. Participants were each assigned a unique, anonymised study ID. All data were entered into Opinio, then extracted into Excel and SPSS for data analysis. Data collection was carried out from September–November 2016.

Non-responders were followed up by study researchers by phone and email, and any manager who declined to complete the survey was not contacted further.

A brief follow-up survey was conducted one year after the initial data collection (October 2017) to ascertain whether any ADUs had opened or closed. Services identified in the original mapping exercise were contacted by phone and email to check they were still operating, and to identify any changes to services.

This survey met the Health Research Authority (HRA) criteria for a service evaluation rather than research, and was approved as such by NoCLOR [15], meaning that the need for ethical approval was waived.

### Analysis

As outlined above, there were two main aims of the survey: i) to establish a typology of ADU models; and ii) to describe current practice in ADUs.

To address aim i), a cluster analysis was carried out. Cluster analysis is a way of grouping units in ways such that those units more similar to each other appear in the same cluster, aiming to minimise variability within clusters, and maximise variability between clusters [16]. There were four stages to the cluster analysis. Firstly, potential grouping variables were identified. These were collated from the questions in the survey, with some grouping variables obtained by the amalgamation of multiple survey questions covering the same topic. Secondly, the expert working group ranked the list of potential grouping variables, ordering them by most to least important in distinguishing different types of ADUs. Thirdly, the five highest ranked grouping variables were included in a cluster analysis (in cases where a grouping variable was considered to have poor quality data available from the survey, it was discarded, and the next highest-ranking variable used instead). Five grouping variables is considered an appropriate number to include in this type of analysis. Fourthly, the cluster analysis was refined, with different models run in order to establish the most appropriate number and composition of groups. The resulting variables were then used in a cluster analysis in SPSS [17]. This process is described in more detail in Additional file 1.

To address aim ii), descriptive data were collated for each survey question, including range, mean and median scores.

## Results

### Cluster analysis

We ran several clustering models with a variety of variables, but each permutation produced a solution with only two clusters. The two resulting typologies aligned with whether the ADU was an NHS service or not. No further typologies were identified in the analysis. As such, the descriptive results characterising ADUs that follow are reported separately for NHS-ADUs and voluntary sector ADUs. The full results of the cluster analysis are described in more detail in Additional file 1.

### Prevalence of ADU services

Forty-five individual ADU services meeting our criteria were identified across England. Of the 45 ADUs identified, 27 (60%) were in NHS Trusts (17 Trusts, 29% of the 58 mental health Trusts in England) eight were joint NHS/voluntary sector services (17%), and ten were voluntary sector services (23%).

**Fig. 1:**
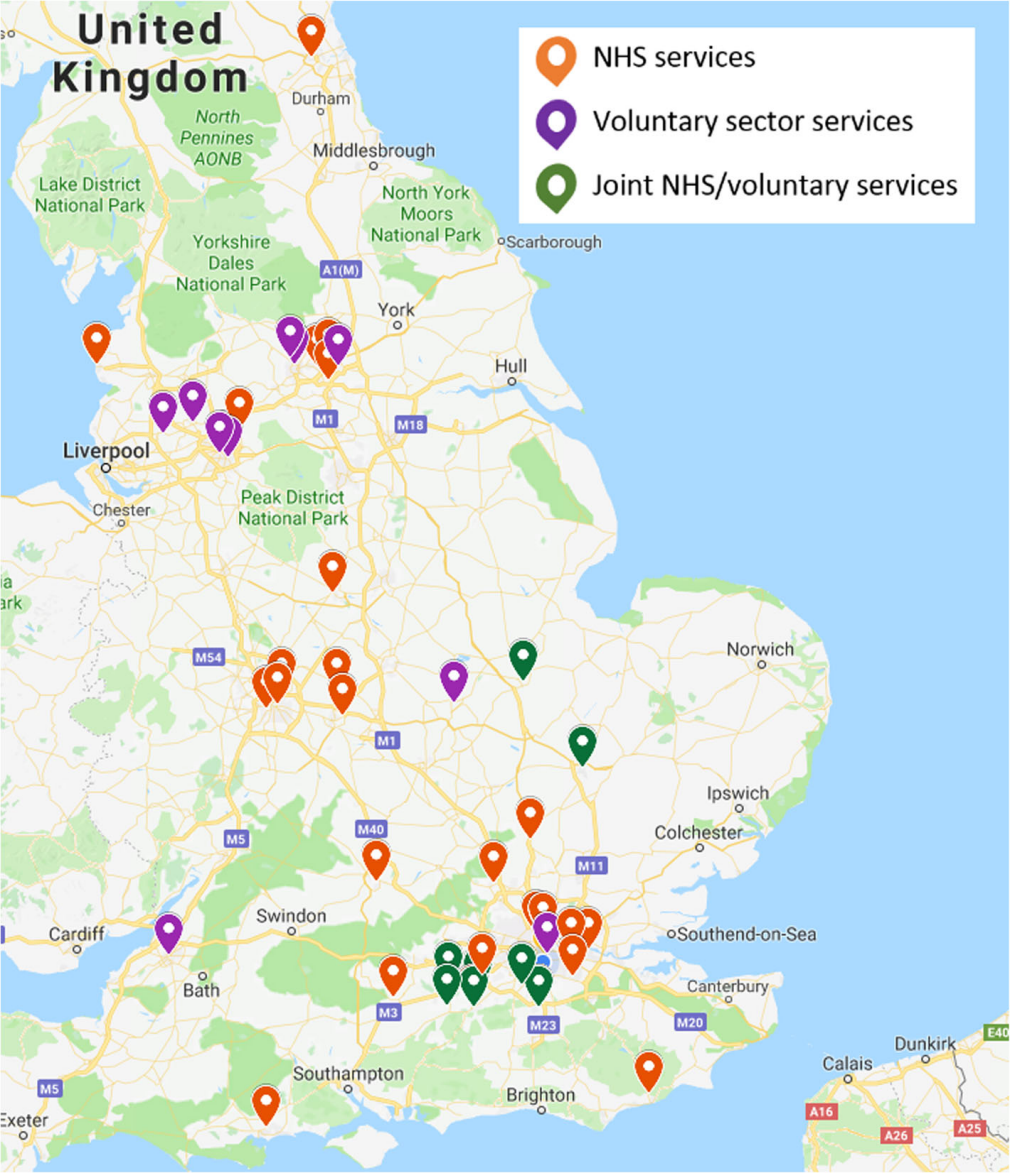
Map of UK ADU services

The geographical locations of the services identified are shown in Fig. 1 below.

In total, 37 of the 45 identified ADU services completed the initial 2016 survey (2 declined, 6 did not respond to multiple requests for information), representing a response rate of 82%. The two ADUs that declined were from the same Trust, but the six that did not respond were from different Trusts and voluntary organisations. Twenty-two NHS services responded to the survey, and 15 joint or voluntary services responded.

Results are reported separately for NHS services (referred to as ‘NHS-ADUs’), and joint and voluntary services (referred to as joint/voluntary). As not every survey respondent answered every question, the denominator is given when reporting each result.

### Location and access

Most NHS-ADUs (*n* = 17/22, 77%) were co-located with other mental health services, with the remainder on independent premises. Most commonly, NHS-ADUs were co-located with CRTs (*n* = 11/17), acute inpatient wards (*n* = 10/17) and Community Mental Health Teams (CMHTs) (*n* = 9/17). Several NHS-ADUs were jointly managed with other acute mental health services (*n* = 13/17). Most NHS-ADUs reported making their own decisions about accepting referrals to their service (‘gatekeeping’) (*n* = 15/22); in other cases, gatekeeping was either joint with a local CRT (*n* = 3), or carried out entirely by another team (*n* = 4).

Very few joint/voluntary services were co-located with CRTs (*n* = 2/15) (both were joint services), with none being jointly managed, and all gatekeeping their own services.

### Purpose of service

In a free-text response to a question asking what the purpose of the service was, eighteen of the 22 NHS-ADUs (82%) stated explicitly that their purpose was to provide an alternative to inpatient admission and/or facilitate early admission from inpatient wards.

All fifteen of the joint/voluntary services expressed their purpose as providing support and/or a safe place for those in mental health crisis. In addition, 11 of the 15 (73%) aimed to provide an alternative to admission to inpatient wards and/or A&E.

### Referrals and discharges

The majority of NHS-ADUs accepted referrals from secondary mental health services, CRTs, and inpatient wards, with some accepting referrals directly from A&E. NHS-ADUs that accepted referrals from other sources, e.g. primary care, or self-referrals, were less common. Nine NHS-ADUs accepted referrals from secondary mental health services only. One NHS-ADU accepted self-referrals from service users or carers. No NHS-ADUs had a completely open access referrals policy. Joint/voluntary services accepted referrals from a wider range of sources, with 6/15 having a completely open access referral policy.

Two NHS-ADUs reported that they rarely refer service users on to other services, because typically they were already using other services as well as the ADU. Two joint/voluntary services also did not refer people on to other services. The remaining services reported a variety of services that they discharged or referred people on to, with the majority of both NHS and joint/voluntary services referring on to secondary mental health services (Table 1).

**Table 1:** Referrals and discharges sources

	Referral		Discharge	
	NHS-ADU n/22 (%)	Joint/voluntary n/15 (%)	NHS-ADU n/20 (%)	Joint/voluntary n/13 (%)
Self/carer	1 (5)	12 (80)	N/A	N/A
CRTs	15 (68)	11 (73)	15 (75)	10 (77)
Crisis Houses	3 (14)	9 (60)	6 (30)	5 (38)
Inpatient wards	18 (82)	8 (53)	17 (85)	6 (46)
Other secondary MH services	16 (73)	12 (80)	17 (85)	12 (92)
GPs	2 (9)	12 (80)	12 (60)	11 (85)
IAPT	3 (14)	10 (67)	N/A	N/A
Other primary care	2 (9)	11 (73)	4 (20)	6 (46)
A&E	9 (41)	13 (87)	N/A	N/A
Police	1 (5)	11 (73)	N/A	N/A
Counselling	N/A	N/A	7 (35)	12 (92)
Welfare advice services	N/A	N/A	7 (35)	8 (62)
Housing services	N/A	N/A	6 (30)	4 (46)

### Client group served by ADUs

Ten of the 22 NHS-ADUs (45%) reported that they had no exclusion criteria. Of the NHS-ADUs with exclusions, 7/12 (67%) would not accept those with a diagnosis of dementia. Other explicit exclusion criteria included a diagnosis of personality disorder (1/12, 8%), brain injury (1/12, 8%), primary alcohol and substance misuse problems (4/12, 33%), learning disability (3/12, 25%), and those unable to engage with the programme offered (1/12, 8%). Only one NHS-ADU (8%) reported that they excluded those who were actively psychotic or unable to keep themselves or others safe.

Of the joint/voluntary services, the only exclusion criteria were being too intoxicated to engage with the service (4/15, 27%), or ‘too high risk’ e.g. actively psychotic (1/15, 6%). Three services of the 15 (20%) also excluded those with very severe learning disabilities that would prevent engagement.

Nineteen of the 22 NHS-ADUs provided data about the age ranges they work with. All these NHS-ADUs worked with service users aged 18–65, apart from five older-age NHS-ADU teams who worked only with adults aged 60 years and older (23%), and one team that worked only with service users aged 24–65 years (5%). Some teams (6/19, 32%) had no upper age limit; 2 teams (11%) would additionally work with people aged 17 upwards.

Two of the 15 joint/voluntary services worked with people aged 16 years and upwards (13%), with the remaining 13/15 working with those aged 18 years and above (87%). Only one service (5%) had an upper age limit, which was 67.

Not all teams responded to questions about service user demographics (which asked for averages over the previous month), but of those that did, Table 2 shows that the average age of people using NHS-ADUs is higher than those using joint/voluntary services. Only three of the joint/voluntary services responded to the question about ethnicity, and two to the question about sexual orientation. Of those that responded, Table 2 shows that the average percentage of service users of different ethnicities and sexual orientations is similar across type of service, with client groups being majority white and heterosexual. These demographics were calculated on the basis of data from the month prior to the survey being completed.

**Table 2:** Service user demographics

	NHS-ADU		Joint/voluntary	
	n/22 teams responding	Median (range) or %	n/15 teams responding	Median (range) or %
Average age	11	48 (28–79)	12	34 (30–46)
Female	16	55%	14	68%
White	13	77%	3	82%
Asian	13	9%	3	8%
Black	13	5%	3	1%
Mixed	13	4%	3	1%
Other	13	4%	3	8%
Heterosexual	10	93%	2	79%

### Length of stay

Six of the 22 NHS-ADUs (27%) had no limit on the maximum length of time a service user could use the service. Those that did (73%) had a limit ranging from 10 days to 6 months, with most (12/16, 75%) being between 6 and 12 weeks. The typical length of time with the service ranged from 15 to 84 days, with the median being 30 days (IQR 23) (18/22 NHS-ADUs responded).

Only three of the 15 joint/voluntary services (20%) put limits on the length of time someone can use the service. Limits ranged from two hours per visit (but no restriction on the number of visits), to three visits per referral (but no restriction on the number of referrals), to 10 days. People using these 15 services typically did so for between 1 and 12 days in a month, with a median of 7 days per month (12/15 services responded).

### Caseload

Of the 18/22 NHS-ADUs responding, the total number of places on the caseload available ranged from 6 to 55 (median 33, IQR 25), with between 3 and 45 service users typically visiting the ADU per day (median 15, IQR 10).

The annual usage also varied substantially among the 17/22 NHS-ADUs that responded. The median number of service users treated in the previous 12 months was 186, IQR 134 (range: 114–2000). The median number of distinct treatment episodes provided was 170, IQR 94 (range: 120–5544).

As the joint/voluntary services do not typically keep a ‘caseload’ in the sense that NHS services do, this survey question was not relevant to them. The median number of people using these services per day was 7, IQR 12 (range: 2–20), and per year the median was 200, IQR 200 (range: 54–400). The median number of periods of care provided by these services was 1874, IQR 3300 (range: 100–6000).

### Opening hours

Most of the 19/21 NHS-ADUs responding reported opening during the working week, in office hours only, with just two running 24-h services. The joint/voluntary services were more varied in their opening hours, with two opening during office hours, 10 opening for some period between 12 pm and 2 am, and three opening from 8 pm to 6 am. None were 24-h services.

### Workforce

Table 3 shows the average number of staff members employed by services in various roles (as well as the range of values given, and number of teams that employed staff in each type of role). NHS-ADUs typically employed more nurses, occupational therapists, and support workers than any other type of staff, and more qualified clinical staff in general; joint/voluntary services employed more peer support workers, and ‘other’ workers, e.g. staff employed to provide general support to people dropping in to such services. In addition to the roles below, four NHS-ADUs reported having a few hours per week from an arts therapist, and one of those also had time from a music therapist and a dance and movement therapist.

**Table 3:** Workforce

	NHS-ADU		Joint/voluntary	
	Total # staff Median (range)	# teams employing staff in role n/22	Total # staff Median (range)	# teams employing staff in role n/15
Nurses	3 (1–10)	18	2 (1–3)	6
Consultant psychiatrists	1 (1–2)	13	0	0
Other medical staff	2 (1–6)	9	0	0
Social workers	1 (1)	2	2 (1–2)	2
Occupational therapists	2 (1–6)	16	0	0
Psychologists	1 (1–2)	11	0	0
Graduate MH workers	1	1	0	0
Pharmacists	1 (1)	6	0	0
Support workers	3 (1–10)	17	3 (2–4)	10
Mental health project workers	0	0	10	1
Crisis recovery workers	0	0	12	1
Administrative staff	1 (1–2)	12	0	0
Peer support workers	1 (1–16)	3	3 (1–13)	6
Counsellors	0	0	2 (1–2)	2
Students	1 (1–7)	8	1	1
Volunteers	1 (1–8)	7	6	1

### Interventions provided

A wide range of interventions were provided by services, but there are no universally provided interventions. A large majority of NHS-ADUs provide support with medication, physical health, relapse prevention, psychological therapies, daily living activities, and one-to-one support. Joint/voluntary services tend not to provide physical or psychological interventions, but all provide one-to-one support, and a large majority provide relapse prevention support. This is shown in Table 4.

**Table 4:** Interventions provided

	NHS-ADU (n/22)	Joint/voluntary (n/15)
	# (%)	# (%)
Medication review, prescription, and dispensing,	19 (86)	0
Medication support and monitoring	19 (86)	6 (40)
Physical health monitoring/ investigation	18 (82)	1 (7)
Self-management/relapse prevention	18 (82)	12 (80)
Advance directives	8 (36)	1 (7)
Psychological therapies	18 (82)	5 (33)
Family work/therapy	7 (32)	1 (7)
Peer-run groups	6 (27)	7 (47)
Carer support groups	9 (41)	3 (20)
Art/drama/music therapy/groups	7 (32)	0
Sports groups	10 (45)	0
Daily living activities	19 (86)	2 (13)
Work experience	2 (9)	5 (33)
Alcohol/substance misuse groups	11 (50)	6 (40)
One-to-one support	20 (91)	15 (100)
Debt/benefits/housing help	15 (68)	8 (53)

### Service user and carer involvement

Table 5 summarises findings from NHS-ADU and joint/voluntary respondents on service user and carer involvement in various aspects of the services. A majority of NHS-ADUs involved service users in staff recruitment, had service user forums, and a large majority sought feedback from service users and, to a lesser extent, carers. Joint/voluntary services had more service user involvement in general, with the majority involving service users and/or carers in management, advisory groups, staff recruitment, feedback (including service users collecting feedback from others), and addressing feedback. A majority also held service user forums and community meetings, and employed peer support workers.

**Table 5:** Service user and carer involvement

	NHS-ADU (n/19)				Joint/voluntary (n/15)			
	SUs		Carers		SUs		Carers	
	#	%	#	%	#	%	#	%
Service management	2	10.5	1	5.3	11	73.3	6	40
Advisory groups	6	31.6	4	21.1	13	86.7	10	66.7
Staff recruitment	12	63.2	5	26.3	12	80	6	40
Staff training	5	26.3	2	10.5	7	46.7	6	40
Delivering interventions	3	15.8	0	0	7	46.7	6	40
Facilitating groups	4	21.1	1	5.3	5	33.3	4	26.7
Feedback about service	17	89.5	13	68.4	14	93.3	11	73.3
Collecting feedback	8	42.1	6	31.6	10	66.7	6	40
Addressing feedback	3	15.8	1	5.3	11	73.3	6	40
Paid positions	2	10.5	0	0	6	40	0	0
Peer support workers	4	21.1	7	36.8	9	60	6	40
Service user/carer forums	12	63.2	4	21.1	10	66.7	6	40
Community meetings	9	47.4	3	15.8	8	53.3	3	20

### Follow up survey

The follow up survey in October 2017 found that five NHS-ADU services had closed down (three in one NHS Trust, the others in two different Trusts), and one had been redesigned to provide a pared down model of ADU care in order to reduce costs. One new NHS Trust had plans to open a pilot ADU, co-located and managed with an existing Crisis Resolution Team (CRT), in early 2018, and, should the pilot site perform well, an additional six ADUs (also alongside existing CRTs) later in 2018. At the time of publishing, this meant there were 23 NHS-ADUs available, covering 14 NHS mental health Trusts (of 58 Trusts in total). All joint/voluntary services identified in the original survey were still operating.

## Discussion

### Main findings

The mapping exercise, which identified 45 ADUs in England, demonstrates that ADUs are not an established part of mental health service provision in most areas. The cluster analysis found evidence of two types of service model: i) NHS services (*n* = 27); and ii) voluntary sector services (including jointly run NHS and voluntary sector services) (*n* = 17). Considering the geographical distribution of services (see Fig. 1), it is clear that large parts of the population have no access to any kind of acute day service as defined by this survey. While the evidence base for ADUs is small, there have been positive findings in previous studies, (i.e. greater symptom improvement and service user satisfaction than for inpatient wards [13]), so it is surprising that ADUs are not more widespread.

The difference between the NHS and joint/voluntary services is quite marked. NHS-ADUs are typically available 10 am-4 pm on weekdays, with a wide range of interventions, a multidisciplinary team including clinically qualified professionals, and service users attending for an average of five weeks. In contrast, joint/voluntary services tend to consist of supportive listening staff without clinical qualifications, who provide brief, one-off support to those in immediate crisis, often in the evening and the early hours of the morning. NHS-ADUs have less service user/carer involvement in paid roles, management, recruitment, and training than the joint/voluntary services. In this regard, NHS-ADUs appear to be involving service users and carers at similar levels to CRTs [8]. While the practical offering of the two types of service are quite different, the explicitly stated purpose of a large majority of both types is as an alternative to inpatient admission. The joint/voluntary services are more often intended as an alternative to A&E, which may explain the difference in daily support offered.

It is notable that there are currently no national (or international) standards for how ADUs should be set up or function, and this perhaps explains the variation evident, for example, in the wide range of interventions offered. Unlike CRTs, Early Intervention Services, and Assertive Outreach Teams, there was no guidance given in the Mental Health Policy Implementation Guide [18] about the composition of NHS-ADUs, resulting in a certain amount of heterogeneity, and no standards or criteria are given by which to assess service functioning. Guidance on the place of ADUs within the acute care pathway is similarly lacking.

The findings of this study are in line with previous research of ADUs, both within England and internationally. For example, a previous survey of psychiatric day hospitals in England found heterogeneity of service provision [19], as did a survey of day hospitals for general psychiatric patients in Germany, England, Poland, the Slovak Republic and the Czech Republic [9], although both studies found that the majority of services aimed to provide an alternative to inpatient admission, similar to the current survey. One aspect we investigated in this survey, the involvement of service users and carers in the management and running of ADUs, is lacking in previous research, and there is little indication from international studies that this issue is addressed elsewhere. It is also unclear from international research whether ADUs are provided by the voluntary sector in other countries, as this survey demonstrates they are in England.

There are similarities between NHS-ADUs and CRTs: both types of team offer a range of interventions, delivered by multidisciplinary teams, as an alternative to admission. The key differences are in the location and timing of contact. Due to service users attending one location during office hours, ADUs are able to offer a wider range of interventions, consistency in terms of the staff service users see, more contact time, and peer support. In comparison, by providing home visits and working shifts, CRT contact time is brief, there is little consistency in which staff member sees which service user, and there is no opportunity for peer support (all of which are well-documented complaints of CRT users [20]). While CRTs offer more flexibility in timing and location of care, and the opportunity for the clinical team to observe a service user’s home environment, for people for whom loneliness, isolation, and lack of activity are a problem, or whose home environment is problematic, ADU care potentially has added benefits than CRT-only care.

In addition to the differences between NHS-ADUs and CRTs, the two ADU models found in this survey (NHS and joint/voluntary services) indicate that there is further complexity in the acute care pathway. The different offerings of NHS and joint/voluntary services may explain the geographical overlap evident in Fig. 1, with joint/voluntary services ‘filling the gaps’ that NHS-ADUs and CRTs do not cover, by providing drop-in services out of office hours. Research into how NHS and voluntary sector services complement each other and work together is currently lacking, though there is a programme of work underway to gain insight into this important area [10].

The follow up survey suggests that NHS-ADU services occupy a precarious position. The closure of five NHS-ADUs in a relatively brief space of time is striking. It implies an unstable environment in which non-mandated services may be seen as easily disposable when there is pressure on resources, despite research evidence suggesting they can be effective [12, 13]. At the same time, the piloting and planned opening of seven new NHS-ADUs in one Trust suggests that the value of such units is recognised by some commissioners, which reflects the importance of providing choices for people in crisis [14].

### Strengths and limitations

There are two main strengths of this survey. The first is the high response rate (82%), meaning that we can take the results to be broadly representative of existing ADUs in England. The second is the inclusion of all services, whether provided by NHS or voluntary sector services, which gives a comprehensive picture of what is available, and where.

There are three key limitations. The first is that because ADUs are not mandated services, lacking a definitive name or model, identifying such services was challenging. While we used a clear and specific definition of the type of team we were interested in, it was frequently the case that one part of a Trust would identify no such teams, and then another source within the Trust would identify a service that clearly met our inclusion criteria. For this reason, and despite the multiple avenues we used to identify teams, it is possible that there are more ADUs in the country than were identified by this survey.

A second limitation was that because we found teams are closing and opening relatively quickly, accurately identifying the number of such services in the country at any one time is challenging.

The third limitation regards the quality of the data obtained in the survey. Many teams did not answer all survey questions. For the joint/voluntary services this was often because the questions were not relevant to them, or, as with questions about ethnicity and sexual orientation, because they do not keep records on these variables, but even among the NHS-ADUs there was some missing data. The survey aimed to be as comprehensive as possible while remaining feasible for busy clinicians to complete, but perhaps a shorter survey would have encouraged a higher completion rate. There is the possibility of social desirability bias from this self-report survey, and that respondents interpreted questions in different ways.

### Research implications

The results of this survey demonstrate the need for further research of these services. While there has been some previous research comparing outcomes for people using ADUs with those using inpatient wards [12], there is little evidence regarding ADUs compared to other non-residential services. The finding [12] that ADUs are as effective as inpatient wards is promising, but it would be helpful to investigate the place and effectiveness of ADUs in the wider acute care context. Understanding how the ADU complements other crisis and community provision by offering choice of support is vital. There is a lack of research considering the acute crisis care system as a whole, and how the range of services available can work together to meet the needs of different people. Investigation of the service user and carer experience of ADUs is also, as far as we can find, entirely lacking, and this is particularly important to rectify. While this survey focussed on ADUs in England, this is an issue of international relevance, and comparison with services in other countries would be helpful.

Given the widespread availability of CRTs as the standard for non-residential crisis care, it is important to know whether ADU provision enhances outcomes for those using acute services. However, the lack of ADU model specification and subsequent heterogeneity of services means that any such research should ensure it considers similar types of service. Research into the different models of ADU care available, and their relative merits in terms of service user outcomes and experiences, would be beneficial, as would a thorough economic evaluation of the costs and benefits of ADUs in comparison to other acute services. The current Acute Day Units as Crisis Alternatives to Residential Care (AD-CARE) study [21] aims to address these issues.

### Implications for policy and practice

A detailed health economic analysis of ADUs would be highly useful for policy-makers and service planners, particularly given the current economic and political climate in the UK. Such an analysis would provide vital information about the best ways to configure services, given the economic pressures NHS Trusts and wider communities find themselves under.

This survey suggests that, on average, there are around 1215 people using NHS-ADUs or voluntary/joint services per day in England. Putting this into context, as of 2017 there were 18,730 mental health inpatient beds in England [22]. Taking the conservative Marshall et al. [12] estimate of the proportion of inpatients suitable for ADUs (23.2%, CI 21.2 to 25.2), this suggests that potentially approximately 3130 additional patients per day could benefit from ADU care. Given the known pressures on beds, frequent out of area placements, and the inherent desirability of offering choice regarding acute care, commissioners and policy-makers should consider the place of ADUs in the acute care pathway. Development of a national policy and implementation of a standard ADU model would mean that such services were less vulnerable to closure during economically challenging periods.

For existing NHS-ADUs, it may be worth considering further how former and current service users and carers can contribute to services, and the ways in which voluntary sector ADUs manage this could be of interest to NHS-ADUs. Greater sharing of best practice between services would certainly be desirable, as the heterogeneity of services suggests that this is currently not a regular occurrence.

## Conclusion

The relatively small number of services found in this English survey suggests that a large proportion of people requiring non-residential daytime support during mental health crises are unable to access it in this way. The results of this survey provide evidence of heterogeneity in the service offered by ADUs in different areas of the country, although there are broad similarities among NHS services when compared to those offered by the voluntary sector. There is some evidence that ADUs are as effective as residential crisis care, but more research is needed that focuses on the economic benefits of such services, outcomes for those using ADUs, their reception by service users and carers, and the experience of those working in these services.

## Supplementary information

**Additional file 1: Table S1.** Ranking of ADU characteristics for cluster analysis. **Table S2.** Model 1 cluster details for variables 1, 4, and 5. **Table S3.** Model 1 cluster details for variable 2 (service provider). **Table S4**. Model 1 cluster details for variable 3 (client group). **Table S5.** Model 2 cluster details for variables 1, 4, 5, and 6. **Table S6.** Model 2 cluster details for variable 3 (client group).

**Additional file 2.** AD-CARE National ADU Survey.

## Data Availability

The datasets generated and/or analysed during the current study are not publicly available due to the small number of ADUs returning data and the potential for individual units being identifiable.
