# Supplementary material for: Adult mental health provision in England: a national survey of acute day units
Source: BMC Health Serv Res. 2019 Nov 21;19:866. doi: 10.1186/s12913-019-4687-8 (PMC6868849; doi:10.1186/s12913-019-4687-8)
Supplement: Supplementary file 1 — Additional file 1: Table S1. Ranking of ADU characteristics for cluster analysis. Table S2. Model 1 cluster details for variables 1, 4, and 5. Table S3. Model 1 cluster details for variable 2 (service provider). Table S4. Model 1 cluster details for variable 3 (client group). Table S5. Model 2 cluster details for variables 1, 4, 5, and 6. Table S6. Model 2 cluster details for variable 3 (client group). [file 12913_2019_4687_MOESM1_ESM.docx]

# Additional file 1

Eight members of a multidisciplinary expert working group ranked 14 variables within the survey identified as relevant to ADU type (Table S1). Variables were ranked by participants using 1 to indicate the most important, and 14 to indicate the least important, meaning that those variables with the lowest total score were considered (by consensus) to be the most important in distinguishing services from each other.

Table S1 Ranking of ADU characteristics for cluster analysis

| Variable | ADU service characteristic | a | b | c | d | e | f | g | h | Total | SD |  |
| --- | --- | --- | --- | --- | --- | --- | --- | --- | --- | --- | --- | --- |
| 1 | Interventions provided | 2 | 3 | 8 | 2 | 7 | 10 | 1 | 1 | 24 | 2.88 |  |
| 2 | Service provider (statutory/ voluntary/joint) | 3 | 1 | 2 | 1 | 5 | 5 | 11 | 6 | 29 | 3.58 |  |
| 3 | Client group served | 1 | 4 | 9 | 4 | 12 | 14 | 2 | 2 | 34 | 4.10 |  |
| 4 | Length of ‘stay’ | 7 | 8 | 3 | 3 | 2 | 11 | 6 | 5 | 34 | 2.27 |  |
| 5 | Staffing (types of staff) | 4 | 9 | 7 | 6 | 3 | 9 | 5 | 4 | 38 | 2.07 |  |
| 6 | Referral sources | 5 | 13 | 1 | 13 | 6 | 13 | 7 | 3 | 48 | 4.63 |  |
| 7 | Opening hours | 8 | 6 | 5 | 5 | 14 | 4 | 4 | 9 | 51 | 3.45 |  |
| 8 | Service user/carer involvement | 14 | 5 | 6 | 8 | 10 | 1 | 10 | 7 | 60 | 3.05 |  |
| 9 | Gatekeeping | 11 | 12 | 12 | 7 | 8 | 12 | 3 | 8 | 61 | 3.25 |  |
| 10 | Co-location of services | 13 | 2 | 13 | 10 | 1 | 2 | 12 | 14 | 65 | 5.47 |  |
| 11 | Size/usage of service | 6 | 7 | 11 | 9 | 9 | 8 | 13 | 10 | 65 | 2.36 |  |
| 12 | Staffing levels (staff FTE*:daily attendance) | 10 | 10 | 4 | 12 | 11 | 7 | 9 | 12 | 68 | 2.75 |  |
| 13 | Joint management of services | 12 | 11 | 14 | 11 | 4 | 3 | 14 | 13 | 79 | 3.45 |  |
| 14 | Discharge destinations | 9 | 14 | 10 | 14 | 13 | 6 | 8 | 11 | 79 | 2.43 |  |
| * Full-Time Equivalent | | | | | | | | | | | | |

Twostep cluster analysis was used to enable inclusion of categorical and continuous variables, and for automatic determination of the optimal number of clusters.

### Model 1

The first model used variables 1, 2, 3, 4, and 5 (as shown in Table S1), and it identified two clusters. Cluster 1 included services that offered a larger number of interventions, a longer average length of ‘stay’, and a more multidisciplinary staff team; they were more likely to be provided by the NHS, and to have restrictions regarding the types of clients taken on. Services in cluster 2 offered a smaller variety of interventions, a shorter ‘stay’, and a less varied multidisciplinary team; they were more likely to be provided by voluntary organisations, and to have fewer restrictions about client groups taken on. The most important distinguishing variable was whether services were provided by statutory bodies (NHS) or voluntary organisations (including joint voluntary/NHS services). The results are shown in Tables S2 (numeric variables), S3 (categorical variable), and S4 (binary variable) below.

Table S2 Model 1 cluster details for variables 1, 4, and 5

|  | | Number of interventions offered from defined list | | Typical length of 'stay' in the team, as reported by the manager | | Number of different staff types in the service | |
| --- | --- | --- | --- | --- | --- | --- | --- |
|  |  | Mean | Std. Deviation | Mean | Std. Deviation | Mean | Std. Deviation |
| Cluster | 1 | 18.31 | 4.936 | 36.81 | 18.552 | 7.19 | 2.689 |
|  | 2 | 9.27 | 5.781 | 7.18 | 3.995 | 2.27 | .647 |
|  | Combined | 14.63 | 6.884 | 24.74 | 20.611 | 5.19 | 3.223 |

Table S3 Model 1 cluster details for variable 2 (service provider)

|  | | Statutory | | Voluntary | | Joint statutory/voluntary | |
| --- | --- | --- | --- | --- | --- | --- | --- |
|  |  | Frequency | Percent | Frequency | Percent | Frequency | Percent |
| Cluster | 1 | 16 | 100.0% | 0 | 0.0% | 0 | 0.0% |
|  | 2 | 0 | 0.0% | 3 | 100.0% | 8 | 100.0% |
|  | Combined | 16 | 100.0% | 3 | 100.0% | 8 | 100.0% |

Table S4 Model 1 cluster details for variable 3 (client group)

|  | | Exclusions | | No exclusions | |
| --- | --- | --- | --- | --- | --- |
|  |  | Frequency | Percent | Frequency | Percent |
| Cluster | 1 | 8 | 80.0% | 8 | 47.1% |
|  | 2 | 2 | 20.0% | 9 | 52.9% |
|  | Combined | 10 | 100.0% | 17 | 100.0% |

### Model 2

The service provider variable (variable 2) could be considered a ‘swamping’ variable (one that has large differences between categories within it, which may overpower weaker, but substantively interesting differences in other variables). As such, a model was run excluding this variable, and including the 6^th^ ranked variable, ‘Number of referral sources’.

Services in cluster 1 offered a larger number of different interventions, a longer average length of ‘stay’, and a more varied multidisciplinary staff team; they accepted referrals from fewer sources, and were more likely to have restrictions regarding the types of clients taken on. Services in cluster 2 offered fewer different types of interventions, a shorter ‘stay’, and a less multidisciplinary team; they accepted referrals from more sources, and had fewer restrictions about client groups taken on.

Once again, even without including the ‘service provider’ variable, the model produced two distinct clusters, with the most important distinguishing variable being whether services were provided by statutory bodies (NHS) or voluntary organisations (including joint voluntary/NHS services). The results are shown in Tables S5 (numeric variables) and S6 (binary variable) below.

Table S5 Model 2 cluster details for variables 1, 4, 5, and 6

|  | | Number of interventions offered from defined list | | Typical length of 'stay' in the team, as reported by the manager | | Number of different staff types in the service | | Number of referral sources | |
| --- | --- | --- | --- | --- | --- | --- | --- | --- | --- |
|  |  | Mean | Std. Deviation | Mean | Std. Deviation | Mean | Std. Deviation | Mean | Std. Deviation |
| Cluster | 1 | 16.67 | 6.721 | 33.11 | 20.522 | 6.67 | 2.951 | 2.44 | 1.688 |
|  | 2 | 10.56 | 5.480 | 8.00 | 3.571 | 2.22 | .667 | 12.11 | 4.567 |
|  | Combined | 14.63 | 6.884 | 24.74 | 20.611 | 5.19 | 3.223 | 5.67 | 5.463 |

Table S6 Model 2 cluster details for variable 3 (client group)

|  | | Exclusions | | No exclusions | |
| --- | --- | --- | --- | --- | --- |
|  |  | Frequency | Percent | Frequency | Percent |
| Cluster | 1 | 10 | 100.0% | 8 | 47.1% |
|  | 2 | 0 | 0.0% | 9 | 52.9% |
|  | Combined | 10 | 100.0% | 17 | 100.0% |
