# Supplementary material for: Adult mental health provision in England: a national survey of acute day units
Source: BMC Health Serv Res. 2019 Nov 21;19:866. doi: 10.1186/s12913-019-4687-8 (PMC6868849; doi:10.1186/s12913-019-4687-8)
Supplement: Supplementary file 2 — Additional file 2. AD-CARE National ADU Survey. [file 12913_2019_4687_MOESM2_ESM.pdf]

Copy of AD-CARE National ADU Survey for Marian

Thank you for participating in the AD-CARE National Survey of Acute Day Units. The information you provide will be extremely helpful in building a comprehensive picture of existing ADU provision across the country. Anonymised results will be shared with those who have taken part, and will be made available more widely via reports and journal articles.

If you have any questions while completing the questionnaire please contact Danielle Lamb on 020 7679 9048 or D.Lamb@UCL.ac.uk.

Location and contact details

Q1: What is the address for your service?

Q2: What is the phone number for your service?

Q3: What is the email address for your service? (Leave blank if your service has no email address)

Q4: If your service has a webpage please enter the URL below. (Leave blank if your service has no webpage)

Q5: Please give the contact details (name, email, phone) for the manager of the service.

Type of service

Q6: Is your service:

- ◯ Public sector - NHS
- ◯ Public sector - local authority
- ◯ Third sector / voluntary / charity
- ◯ Social enterprise
- ◯ Private
- ◯ Other

If you have chosen "other", please specify:

Funding

Q7: How is your service funded? (If your service is part of the NHS, please include the Clinical Commissioning Group that commissions your service)

Purpose of service

Q8: Please describe briefly the purpose of your service. (200 words max)

Record system

Note: if you have answered/chosen item [2, 3, 4, 5, 6] in question 6, skip the following question

Q9: Which record system do you use in your Trust? (e.g. RiO)

Co-location of services

Q10: Is your service co-located with any other mental health services?

◯ Yes ◯ No

Note: if you have answered/chosen item [2] in question 10, skip the following question

Q11: Which services are you co-located with? (Please select all that apply)

☐ Acute inpatient ward
☐ Crisis Resolution and Home Treatment Team
☐ Crisis House
☐ Community Mental Health Team
☐ Other mental health service providing longer-term community care
☐ Other

If you have chosen "other", please specify:

Q12: Are there any joint management arrangements for your service? (E.g. Service Manager also manages the CRT or Crisis House)

◯ Yes ◯ No ◯ Not applicable

Q13: Please explain the joint management structure for your service.

Q14: Are there any 'gatekeeping' arrangements for the places available in your service? For example, do you decide who is offered a place in your service, or does another team (e.g. the local Crisis Resolution and Home Treatment Team) offer places to people?

Q15: Can service users staying on acute wards use your service?

○ Yes ○ No ○ Not applicable

Q16: Can service users on leave from acute wards use your service?

○ Yes ○ No ○ Not applicable

Q17: Does your service facilitate early discharge from wards?

○ Yes ○ No ○ Not applicable

Q18: Do you regularly manage service users' care in conjunction with other services? (E.g. having joint meetings with the service user and members of staff from another service, having joint management meetings with another service, etc.)

☐ Yes ☐ No ☐ Not applicable

If you have chosen "other", please specify:

Note: if you have answered/chosen at least one of the following items: [2, 3] in question 18, skip the following question

Q19: Which services do you regularly work with to manage service users' care?

☐ Community Mental Health Team
☐ Crisis Resolution and Home Treatment Team
☐ Third sector organisations
☐ Other
☐ Psychiatric Liaison Team
☐ Crisis House
☐ Not applicable

If you have chosen "other", please specify:

Note: if you have answered/chosen at least one of the following items: [2, 3] in question 18, skip the following question

Q20: Which of the following ways do you jointly manage service users' care with other services?

- ☐ Joint meetings with service users and staff from other services
- ☐ Joint management meetings with staff from other services
- ☐ Other

If you have chosen "other", please specify:

Q21: Is transport provided to help service users attend your service?

○ Yes ○ No

Note: if you have answered/chosen item [2] in question 21, skip the following question

Q22: Please give details of the transport provided to service users to help them access your service.

Q23: Are you aware of any specific problems for service users accessing your service regarding the location?

○ Yes ○ No

Note: if you have answered/chosen item [2] in question 23, skip the following question

Q24: Please give details of the specific problems service users face in accessing your service regarding the location.

Interventions provided

Q25: What can people do when they visit your service? (Please select all that apply)

- ☐ Attend social groups
- ☐ Chat with other service users
- ☐ Help make lunch/other meals
- ☐ Play table tennis/pool/other games
- ☐ Attend therapy sessions
- ☐ See a specialist for a support meeting
- ☐ Give/receive peer support
- ☐ Informal chat with staff
- ☐ Use a computer
- ☐ Gardening
- ☐ Attend review meetings
- ☐ Other

If you have chosen "other", please specify:

Q26: Which of the following interventions does your service provide? (Please select all that apply)

- ☐ Medical review
- ☐ Prescriptions
- ☐ Dispensing medication
- ☐ Monitoring medication
- ☐ Support with medication adherence
- ☐ Monitoring of medication side-effects
- ☐ Physical health monitoring
- ☐ Physical health investigations
- ☐ Cognitive behavioural therapy
- ☐ Other psychological therapies
- ☐ Supported self-management (e.g. WRAP)
- ☐ Written relapse prevention plans
- ☐ Written advance directives
- ☐ Psychological therapies in groups
- ☐ Psychological therapies 1:1
- ☐ Family therapy
- ☐ Family work
- ☐ Peer-run groups
- ☐ Carers support groups
- ☐ Stress management for carers
- ☐ Art psychotherapy
- ☐ Drama psychotherapy
- ☐ Music psychotherapy
- ☐ Sports groups
- ☐ Daily living activities support (e.g. cooking)
- ☐ Work experience
- ☐ Alcohol/substance misuse groups/advice
- ☐ One-to-one support
- ☐ Debt management

☐ Benefits advice
☐ Help resolving immediate housing problems (e.g. problems with tenancy, arranging cleaning etc.)
☐ Not applicable
☐ Other

If you have chosen "other", please specify:

Referrals

Note: if you have answered/chosen item [1] in question 6, skip the following question

Q27: Please indicate which sources service users come to you from, and, if know, the average number per month from each source. If service users come to you from sources not listed below, please indicate these (as well as the average number per month from each) in the free text box below.

Service	Referrals accepted	Average number of referrals received per month
Service users who have used the service previously	☐	
Carers/family/friends of people who have used the service previously	☐	
Service users who have not used the service previously	☐	
Carers/family/friends of people who have not used the service previously	☐	
Acute psychiatric ward	☐	
Crisis Resolution and Home Treatment Team	☐	
Crisis assessment service (if separate from CRHTT)	☐	
Crisis House	☐	
Other Secondary mental health service (e.g. Early Intervention Service)	☐	
IAPT	☐	

GP	☐	
Other Primary Care	☐	
Police	☐	
A&E	☐	
Other sources	☐	

Note: if you have answered/chosen item [2, 3, 4, 5, 6] in question 6, skip the following question

Q28: Please indicate which sources you accept referrals from, and (where relevant) the average number of referrals accepted per month from each source. If you accept referrals from sources not listed below, please indicate these (as well as the average number of referrals accepted per month from each) in the free text box below.

Service	Referrals accepted	Average number of referrals received per month
Self-referral by service users who have used the service previously (or their carers)	☐	
Referral by carers/family/friends of people who have used the service previously	☐	
Self-referral by service users who have not used the service previously (or their carers)	☐	
Referral by carers/family/friends of people who have not used the service previously	☐	
Acute psychiatric ward	☐	
Crisis Resolution and Home Treatment Team	☐	
Crisis assessment service (if separate from CRHTT)	☐	

Crisis House	☐	
Other Secondary mental health service	☐	
IAPT	☐	
GP	☐	
Other Primary Care	☐	
Police	☐	
A&E	☐	
Other sources	☐	

Moving on from your service

Q29: Please indicate which services you are able to refer service users on to , and (where relevant) the average number of service users per month for each. If you are able to refer on to sources not listed below, please indicate these (as well as the average number of service users per month for each) in the free text box below.

Service	Discharge destination	Average number of discharges per month
Acute psychiatric ward	☐	
Crisis Resolution and Home Treatment Team	☐	
Crisis House	☐	

NHS Community mental health services (e.g. CMHT, EIS, Recovery team etc.)	☐	
GP	☐	
Other Primary Care	☐	
Housing services	☐	
Welfare rights advice	☐	
Counselling	☐	
Other sources	☐	

Q30: What proportion of service users do you estimate return to your service within three months of leaving?

Q31: Are there any kinds of interventions to bridge the gap some services users experience between leaving your service and taken on by another service (e.g. a CMHT)? For example, peer support is used in some areas to maintain continuity of care.

○ Yes ○ No ○ Not applicable

Note: if you have answered/chosen item [2, 3] in question 31, skip the following question

Q32: Please tell us about interventions in place to bridge the gap between your service and any that you discharge to.

Client group served

Q33: Do you exclude any groups of people, who have a mental health problem, from using your service?

- ☐ No exclusions
- ☐ People with a personality disorder
- ☐ People with a learning disability
- ☐ People with dementia
- ☐ People with alcohol and substance misuse problems
- ☐ Other

If you have chosen "other", please specify:

Q34: Please outline the catchment area your service covers (if relevant).

Q35: Do you have any other criteria people must meet in order to use your service?

Duration of care

Q36: Do you have a maximum length of time people can use your service for? E.g. 6 weeks, 3 months, etc.

○ Yes ○ No

Note: if you have answered/chosen item [2] in question 36, skip the following question

Q37: What is the maximum length of time people can use your service for?

Opening hours

Q38: What are the opening hours of your service?

Service capacity

Q39: How many places are available in your service? (I.e. how many people can physically come into the service at one time)

Number	
Not applicable	☐

Q40: How many people can you have on your caseload/books at one time?

Number	
Not applicable	☐

Q41: Do you have a waiting list for your service? If so, how many people are currently on the waiting list?

Waiting list available	☐	Number on waiting list	
Not applicable	☐		

Q42: What is the average waiting time once a service user has been referred to you until they first attend your service?

Hours	
Days	
Weeks	
Not applicable - immediate access	☐
Not applicable - other	

Service usage

Q43: How many people have used your service in the past 12 months? (Please count each service user only once, even if they have been with your service for multiple periods of care)

Q44: How many periods of care have you provided in the past 12 months? (Please count each period of care, i.e. repeat users should be counted multiple times)

Q45: How long do service users typically stay with your service? (Please give answer in number of days, averaged over all service users seen in the past month)

Q46: How many people, on average, attend your service per day?

Q47: What is the average number of people on your caseload/books per day?

Q48: What is your Did Not Attend (DNA) rate? (Please give the average number of DNAs per day)

Service user demographics

Q49: What is the age range of the service users accepted onto your caseload?

Q50: What was the average age of service users on your caseload, in the past month?

Average age	
Unknown	☐

Q51: What percentage of your caseload were female and male, in the past month?

% Female	
% Male	
Unknown	☐

Q52: Please enter the percentage of your caseload that identify as the following ethnic groups, for the past month.

White	
Mixed/multiple ethnicity	
Asian/Asian British	
Black/Black British	
Other Ethnic Group	
Unknown	☐

Q53: What percentage of your service users identify as the following:

Heterosexual or straight	
Gay or lesbian	
Bisexual	
Other	
Prefer not to say	
Unknown	☐

Care cluster

Q54: Please enter the percentage of service users on your caseload in each of the care clusters below, for the past month, if known. (Please enter a number only)

Cluster	% of service users
1 Common Mental Health Problems (low severity)	
2 Common Mental Health Problems (Low Severity with greater need)	
3 Non Psychotic (Moderate Severity)	
4 Non-psychotic (Severe)	
5 Non-psychotic Disorders (Very Severe)	
6 Non-psychotic Disorder of Over-valued Ideas	
7 Enduring Non-psychotic Disorders (High Disability)	
8 Non-Psychotic Chaotic and Challenging Disorders	
9 Blank cluster	
10 First Episode Psychosis	
11 Ongoing Recurrent Psychosis (Low Symptoms)	
12 Ongoing or recurrent Psychosis (High Disability)	
13 Ongoing or Recurrent Psychosis (high symptom and disability)	
14 Psychotic Crisis	
15 Severe Psychotic Depression	

16 Dual Diagnosis	
17 Psychosis and Affective Disorder – Difficult to Engage	
18 Cognitive Impairment (Low Need)	
19 Cognitive Impairment or Dementia Complicated (Moderate Need)	
20 Cognitive Impairment or Dementia Complicated (High Need)	
21 Cognitive Impairment or Dementia (High Physical or Engagement)	
Not applicable	☐
Unknown	☐

Staff groups

Q55: Please indicate how many staff you have in each of the following groups. Please note the number of individuals, and the number of full time equivalent (FTE) staff roles this equates to. E.g. Two people working 1/2 time each would count as 1 FTE.

Staff group	Number of staff	FTE roles
Nurse		
Consultant psychiatrist		
Other medical staff		
Social worker		
Occupational therapist		
Clinical psychologist		
Graduate mental health worker		
Pharmacist		
Support worker		
Admin staff		
Peer support worker		

Counsellors		
Students		
Volunteers		
Other		

Q56: Do you perceive a need for additional staff within your service?

○ Yes ○ No

Note: if you have answered/chosen item [2] in question 56, skip the following question

Q57: Please indicate how many additional staff from each of the groups below you feel your service needs.

Staff group	Number (FTE)
Nurse	
Consultant psychiatrist	
Other medical staff	
Social worker	
Occupational therapist	
Clinical psychologist	
Graduate mental health worker	
Pharmacist	
Support worker	
Counsellors	
Admin staff	
Peer support worker	
Other	

Service user involvement

Q58: Are service users involved with any of the following? (Please select all that apply)

- ☐ Service management
- ☐ Staff recruitment
- ☐ Delivering interventions
- ☐ Invited to give feedback about the service
- ☐ Acting on feedback or complaints
- ☐ Advisory or consultative groups
- ☐ Staff training
- ☐ Facilitating groups
- ☐ Collecting service user or carer feedback
- ☐ Paid positions

- [ ] Peer support workers
- [ ] Community meetings
- [ ] Other
- [ ] Service user or carer forums
- [ ] None of the above

If you have chosen "other", please specify:

Carer involvement

Q59: Are carers involved with any of the following? (Please select all that apply)

- [ ] Service management
- [ ] Staff recruitment
- [ ] Delivering interventions
- [ ] Invited to give feedback about the service
- [ ] Acting on feedback or complaints
- [ ] Service user or carer forums
- [ ] None of the above
- [ ] Advisory or consultative groups
- [ ] Staff training
- [ ] Facilitating groups
- [ ] Collecting service user or carer feedback
- [ ] Paid positions
- [ ] Community meetings
- [ ] Other

If you have chosen "other", please specify:

Service development

Q60: What is the annual budget for your service?

Q61: How long has your service been operating for?

Months	
Years	

Q62: Have there been any changes in your service's role or structure during this time?

◯ Yes ◯ No

Note: if you have answered/chosen item [2] in question 62, skip the following question

Q63: How has your service changed in role or structure since it was first set up?

Q64: Are there any current service improvement initiatives being undertaken in your service?

◯ Yes ◯ No

Note: if you have answered/chosen item [2] in question 64, skip the following question

Q65: Please tell us about these initiatives.

Q66: What are your priorities for maintaining or improving your service?

Q67: Is there anything else you would like to tell us about your service? If so please use the box below.
